# Kin Selection and the Evolution of Social Information Use in Animal Conflict

**DOI:** 10.1371/journal.pone.0031664

**Published:** 2012-02-22

**Authors:** Christopher C. M. Baker, Sasha R. X. Dall, Daniel J. Rankin

**Affiliations:** 1 Department of Organismic and Evolutionary Biology, Harvard University, Cambridge, Massachusetts, United States of America; 2 Centre for Ecology and Conservation, Biosciences, College of Life and Environmental Sciences, University of Exeter, Penryn, Cornwall, United Kingdom; 3 Institute of Evolutionary Biology and Environmental Studies, University of Zürich, Zürich, Switzerland; 4 Swiss Institute of Bioinformatics, Quartier Sorge, Bâtiment Génopode, Lausanne, Switzerland; University of Sheffield, United Kingdom

## Abstract

Animals often use social information about conspecifics in making decisions about cooperation and conflict. While the importance of kin selection in the evolution of intraspecific cooperation and conflict is widely acknowledged, few studies have examined how relatedness influences the evolution of social information use. Here we specifically examine how relatedness affects the evolution of a stylised form of social information use known as eavesdropping. Eavesdropping involves individuals escalating conflicts with rivals observed to have lost their last encounter and avoiding fights with those seen to have won. We use a game theoretical model to examine how relatedness affects the evolution of eavesdropping, both when strategies are discrete and when they are continuous or mixed. We show that relatedness influences the evolution of eavesdropping, such that information use peaks at intermediate relatedness. Our study highlights the importance of considering kin selection when exploring the evolution of complex forms of information use.

## Introduction

Animals frequently rely on information about conspecifics in making decisions regarding mate choice, cooperation or conflicts over resources [1], [2], [3]. Such information can be provided ‘intentionally’, as in the case of signalling, or inadvertently, such as when an individual's actions or their consequences may be observed by others [2], [3], [4]. Gathering information about conspecifics can both help to promote cooperation, as in the case of image scoring [5], [6], or help to resolve conflicts, as in the case of eavesdropping [7]. In image scoring, individuals react to observed cooperation between others by offering help to partners that were previously seen helping others, and refusing help to partners that were unhelpful [8]. A similar situation occurs in eavesdropping, where individuals observe conflicts and use this information by fighting individuals that lost their last encounter and avoiding fights with individuals that won [7]. This type of social information use has been demonstrated in animals (e.g. [9]) and represents a heuristic that may improve an individual's expected outcome from an interaction, but with smaller investments in cognitive capacity and information-gathering than more accurate decision rules, such as full Bayesian updating over a series of interactions.

Almost all social interactions inherently involve interactions with related individuals [10], [11]. Such interactions can help to promote cooperation and resolve conflict between individuals [12], [13], [14]. For example, in the case of animal conflict, it has been shown that higher relatedness between partners favours less escalation (i.e. playing ‘dove’) in the classic hawk-dove game [15]. However, models of social information use in animal conflict and cooperation generally ignore the potential impact that interactions between relatives can have on the evolution of a given behaviour. In a previous model of eavesdropping [7], it was assumed that interactions take place randomly between individuals in an infinitely large population. However, real populations often exhibit population structure: interactions do not take place randomly but rather take place between relatives more commonly than would be predicted by chance in a well-mixed population. Such structure can arise through kin recognition, territorial behaviour, or as a result of limited dispersal. In structured populations, selection should favour individuals that help or avoid conflict with relatives, as well as those that are able to make the most of their interactions with non-relatives. Monitoring simple social cues through eavesdropping potentially addresses both of these criteria, by allowing players to condition their behaviour on information about individual opponents.

In this paper we examine how relatedness affects the evolution of information use in an eavesdropping game. We model the classic hawk-dove game [16] with eavesdropping [7] and with interactions between relatives. We use two variants of the model – one with discrete strategies and one with continuous strategies – as these variants are known to yield different results in the game without eavesdropping [15]. The discrete strategies version is a direct extension of a previous model of eavesdropping by Johnstone [7], in which hawk, dove and eavesdropping phenotypes each arise from separate genotypes. In the continuous strategies version, each genotype gives rise to a proportion of individuals with the eavesdropping phenotype, a proportion with the hawk phenotype, and a remaining proportion with the dove phenotype. Our results suggest that eavesdropping will be most favoured at intermediate relatedness and highlight the importance of considering population structure in studying animal conflict and the evolution of social information use.

## Model and Results

Our model for the evolution of eavesdropping among related individuals is based on the two-player hawk-dove game [17], [18]. Animals frequently compete for resources with each other [19], and the hawk-dove game is a well-studied approach to examining these interactions. It has also been used previously to explore the evolution of cooperation [16], [20]. We model two variants of the eavesdropping game played among relatives: one with discrete strategies, and one with continuously variable strategies. In any given interaction, each of the two players chooses between the actions *hawk* and *dove*. If both select *hawk* then each wins the resource value *v* with probability 0.5 but otherwise bears a cost of fighting *c*, so that the expected payoff is (*v*–*c*)/2 with *c*>*v*>0. (The analysis of the case *v*>*c*>0, i.e. the prisoner's dilemma with eavesdropping, is not dissimilar but omitted here for brevity.) If both select *dove* then each wins the resource with probability 0.5 without bearing the costs of fighting, giving an expected payoff of *v*/2. If one player chooses *hawk* and the other *dove*, then the hawk wins the resource value *v* with certainty, while the dove receives 0; both hawk and dove in this scenario avoid the cost of fighting *c*. Note that these expected payoffs would be the same under the common alternative formulation of the hawk-dove game in which the resource is split evenly between a pair of hawks or a pair of doves rather than being randomly assigned; but our eavesdropping strategy assumes the presence of a clear winner to provide a potential source of information to eavesdropping observers, as described below.

We assume an infinite population, where each individual plays a large number of interactions over its lifetime before reproducing clonally. The reproductive success or fitness of an individual is proportional to the average payoff across all interactions during its lifetime. There are no repeated interactions, but we allow for the possibility of eavesdropping: an eavesdropper plays *dove* in any interaction where the opponent's prior encounter was perceived as a win, and otherwise plays *hawk*.

### Discrete strategies model

The discrete strategies model envisages three distinct genotypes, each corresponding to a different strategy that may be thought of as a phenotype. An individual with the hawk genotype always plays the action *hawk*; a dove always plays *dove*; and an eavesdropper plays the conditional eavesdropping strategy, which may dictate either *hawk* or *dove* in any given encounter. Johnstone's original model [7] assumes that opponents are drawn randomly from the whole population, so that genotypes encounter one another in proportions determined by their frequencies in the population. We allow for non-random assortment by introducing relatedness as an exogenous parameter reflecting, for example, limited dispersal. The relatedness *r* measures the probability that a player's opponent has the same genotype as the player, relative to the probability of obtaining the same genotype in a randomly drawn member of the population. This is a standard method of introducing relatedness in simple game-theoretical models (e.g. [15], [21], [22]). Thus, an individual with genotype *i* plays another type *i* individual with probability
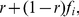
(1)and plays an opponent of type *j*≠*i* with probability
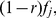
(2)where *f_i_* and *f_j_* are the frequencies of genotypes *i* and *j* in the population. When *r* = 1, pairs of players always have the same genotype; when *r* = 0, players interact with each genotype in proportion to the population frequencies. Although relatedness may, in principle, be negative (e.g. [23], [24]), we restrict our analysis to 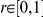. Note that *r* measures assortment at the level of the genotype (i.e. hawk, dove or eavesdropper) rather than action (i.e. *hawk* or *dove*) – for example, an eavesdropper meets another eavesdropper with probability *r*+(1–*r*) *f_E_*, but in a given interaction the two may or may not play the same action, since each player's action depends on the outcome of its opponent's previous encounter.

The probability *p_i_* that a type *i* individual won its last encounter settles down after relatively few iterations of the game, and is given by
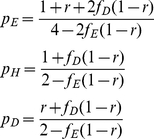
(3)with genotype frequencies *f_E_*+*f_H_*+*f_D_* = 1. The derivation of these and other expressions is provided in more detail in Material S1.

**Figure 1:**
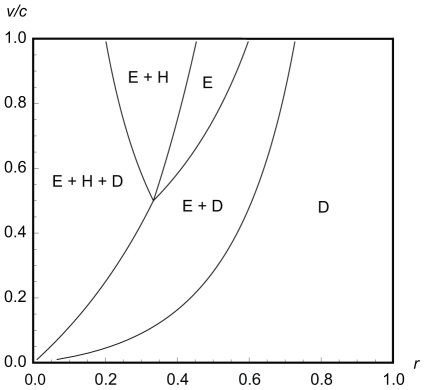
Phenotypes in equilibrium in the discrete strategies model. Labels indicate genotypes with positive equilibrium frequencies under error-free eavesdropping (α = 1) with E = eavesdroppers, H = hawks and D = doves.

**Figure 2:**
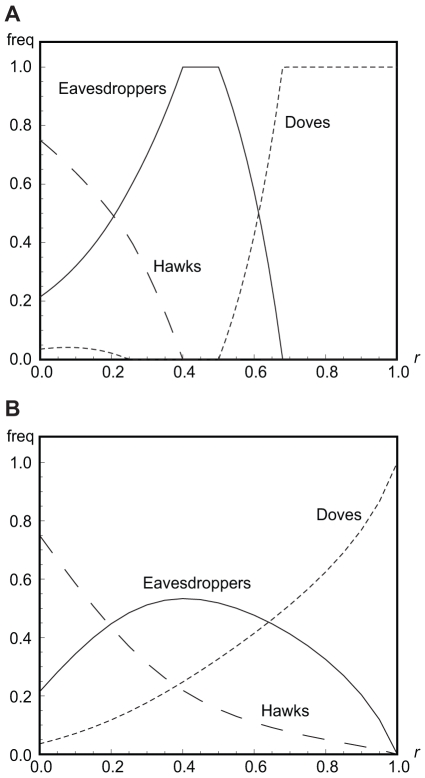
Equilibrium phenotype frequencies for *v*/*c* = 0.75 under error-free eavesdropping (α = 1). Panel A: results for the discrete strategies model. Panel B: results for the continuous strategies model.

**Figure 3:**
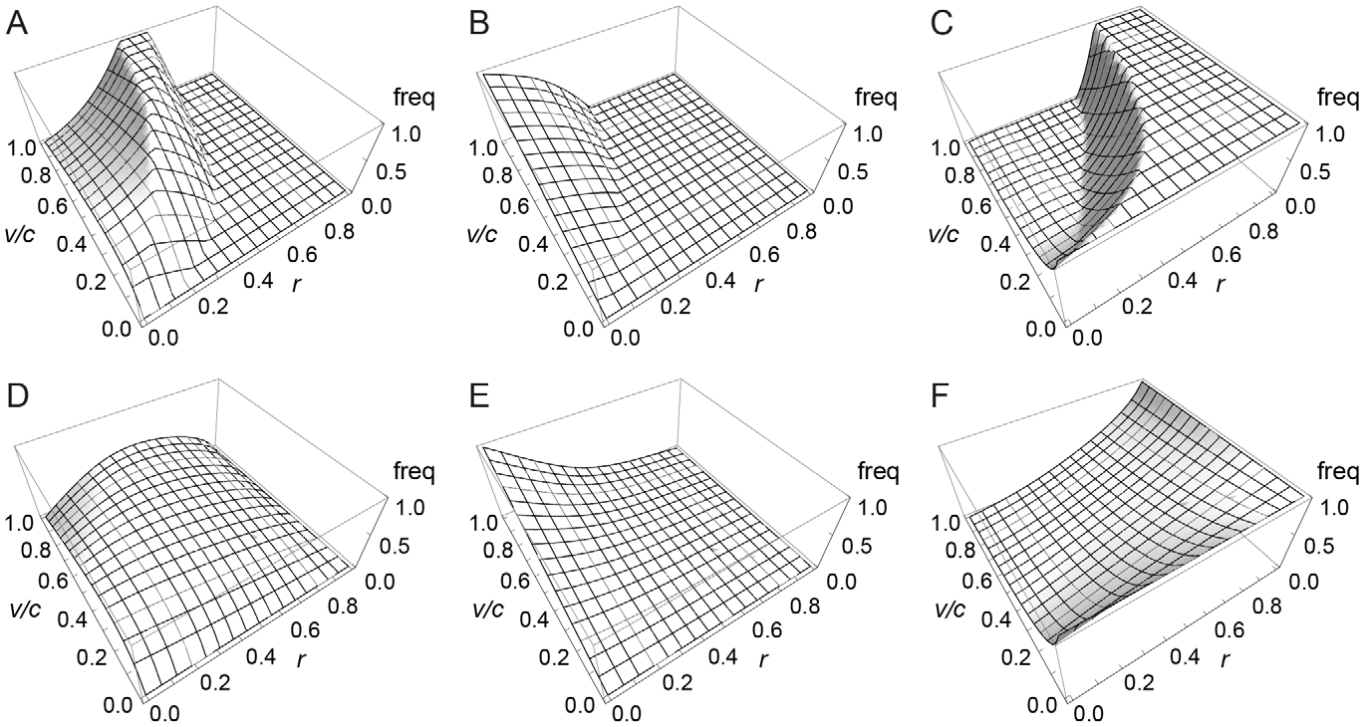
Equilibrium phenotype frequencies under error-free eavesdropping (α = 1). Panels A, B, C: frequencies of eavesdroppers, hawks and doves in the discrete strategies model. Panels D, E, F: frequencies of eavesdroppers, hawks and doves in the continuous strategies model.

We use these probabilities to determine each genotype's average payoff as a function of the *f_i_*. We assume no mutation or drift, and allow the frequencies of the eavesdropper, hawk and dove genotypes to evolve according to standard continuous replicator dynamics [25], [26]. Solving for the frequencies that give equal fitness to the three genotypes gives the following long-run equilibrium frequencies:
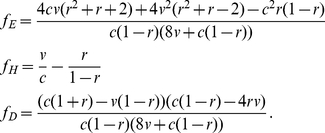
(4)When *r* = 0, the model is identical to that in [7]. For positive *r*, all three genotypes still coexist stably, at frequencies given by (4), as long as
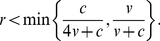
(5)But if (5) does not hold, then (4) gives frequencies outside the range [0,1], implying that one or more of the genotypes will be driven to extinction or fixation. For *v*/*c*>0.5, eavesdroppers and hawks coexist stably, with doves driven towards extinction over time, if
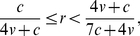
(6)and eavesdroppers go to fixation if
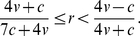
(7)If 
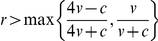
, eavesdroppers and doves coexist stably, with hawks driven towards extinction over time, if
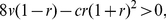
(8)otherwise doves go to fixation. Figures 1, 2 and 3 summarise these equilibria as a function of *r* and *v*/*c*. A more detailed derivation and description of these results is shown in Material S1.

If eavesdroppers make errors in determining the outcomes of their adversaries' prior encounters, we find that eavesdropping peaks at a lower level of relatedness than in the absence of errors. We model errors by introducing an ‘accuracy’ parameter 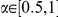 describing the probability that an eavesdropper correctly perceives an adversary's prior outcome (formal results not shown but available from the authors on request). With probability α, an eavesdropper perceives a win and plays *dove* when the adversary's prior outcome was truly a win, and perceives a loss and plays *hawk* when the prior outcome was truly a loss; with probability (1 – α), the eavesdropper perceives a win and plays *dove* when the prior outcome was actually a loss, and perceives a loss and plays *hawk* when the prior outcome was actually a win. At low relatedness, errors make eavesdropping more attractive if fight costs are low since the population is dominated by eavesdroppers and hawks, and errors allow an eavesdropper to avoid some escalated fights when playing another eavesdropper, although this is partly offset by greater average fight costs when encountering a hawk. If fight costs and/or relatedness are high, eavesdroppers and doves dominate the population, and errors increase the rate of escalated fights among pairs of eavesdroppers, thus selecting against eavesdropping.

**Figure 4:**
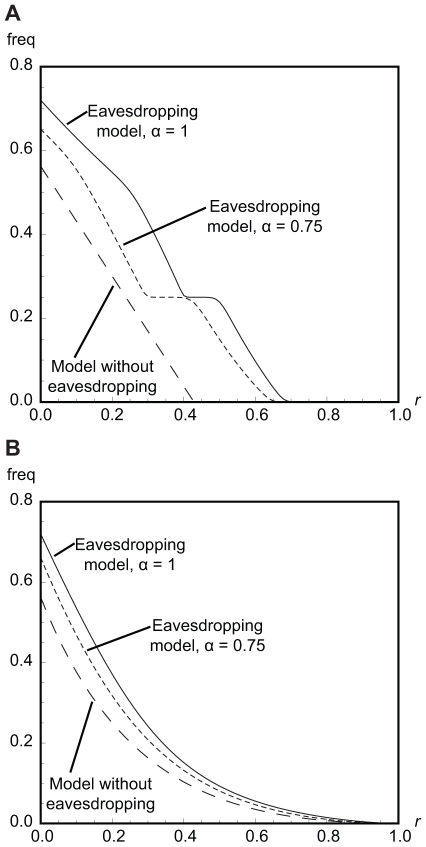
Frequency of escalated conflicts for *v*/*c* = 0.75. Escalated conflicts are interactions in which both players play the action *hawk*. Panel A: frequencies for the discrete strategies model. Panel B: frequencies for the continuous strategies model.

In the model with eavesdropping, individual aggression (i.e. *hawk* actions) and escalated conflicts (i.e. *hawk*-*hawk* encounters) *generally* occur at higher frequency than in the model without eavesdropping (Figure 4; see also Material S1). As in previous work [7], there is an incentive for more aggression than would otherwise occur, since this improves a player's chance of winning future encounters with eavesdroppers. However, at low relatedness, the frequency of escalated conflict is lower than would be expected given the frequency of individual aggression, essentially since eavesdroppers are able to avoid conflict against aggressive opponents. At intermediate relatedness, this is more than offset by the fact that aggressive individuals interact among themselves more often than would be expected by chance, so that the frequency of escalated conflict is higher than might be expected (see Material S1). At high relatedness, doves go to fixation and there is no aggression or escalated conflict at all.

In contrast to the model of Johnstone [7], the discrete strategies model with relatedness *can* produce lower frequencies of individual aggression (i.e. *hawk* actions) and escalated conflict (i.e. *hawk*-*hawk* encounters) than the model without eavesdropping. This happens within a narrow range of parameters, when fighting is not very costly (*v*/*c* close to 1), eavesdropping is very error prone (α close to 0.5) and there is moderate population structure as captured by *r*. This combination of parameters produces an equilibrium with fewer hawks than in the model without eavesdropping, i.e. [15]. In the model with eavesdropping, however, individual aggression also includes any eavesdroppers that play the *hawk* action. With α close to 0.5, eavesdroppers are essentially choosing randomly between *hawk* and *dove* in each interaction, so individual aggression is the frequency of hawks plus approximately half of the frequency of eavesdroppers. Escalated aggression in the model without eavesdroppers is just the frequency of hawk-hawk interactions but, in the model with eavesdroppers, interactions in which eavesdroppers play the *hawk* action must also be taken into account. The frequencies of individual aggression and escalated conflict are lower than in the model without eavesdropping provided that eavesdroppers are sufficiently abundant in equilibrium, and that low costs of fighting ensure that hawks are abundant in the non-eavesdropping model.

### Continuous strategies model

In the continuous strategies model, a genotype *i* displays phenotypic plasticity as described by two parameters, 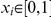 and 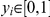. We assume phenotypic noise at birth such that, when each genotype *i* individual is born, it becomes a hawk, dove or eavesdropper at random but with probabilities determined by its genotype (*x_i_*,*y_i_*). Of the genotype *i* individuals, a proportion *x_i_* takes on an eavesdropper phenotype for life and thus plays the conditional eavesdropping strategy in every interaction. A proportion (1-*x_i_*)*y_i_* takes on the hawk phenotype and thus always plays the action *hawk*; likewise a proportion (1-*x_i_*)(1-*y_i_*) takes on the dove phenotype and so always plays the action *dove*. Since the relevant evolutionary dynamics in our model take place at the genotype level, the fitness of genotype *i* is the weighted average fitness of its three phenotypes, where phenotype fitness is again measured as the average across all interactions during an individual's lifetime.

As in the discrete strategies model, we allow for non-random assortment among genotypes arising from, say, limited dispersal, by permitting non-zero relatedness. The exogenous relatedness parameter *r* measures the probability that a player's opponent has the same genotype as the player, relative to the probability of drawing the same genotype randomly from the population. An individual with genotype *i* plays another type *i* individual with probability *r*+(1−*r*) *f_i_*, and plays an opponent of type *j*≠*i* with probability (1−*r*) *f_j_*, where *f_i_* and *f_j_* are the frequencies of genotypes *i* and *j* in the population. In the continuous strategies model, genotypes *i* and *j* will be a resident genotype close to fixation and a mutant genotype at low frequency, since our equilibrium analysis will concentrate on finding genotypes that cannot be invaded by ‘nearby’ mutants. We again restrict attention to 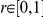. Note that assortment takes place at the genotype level, and when two players have the same genotype this means that they share the same values for *x* and *y*. However, at the time of the interaction, their phenotypes are already determined as eavesdropper, hawk or dove, and the two players may or may not share the same phenotype. Additionally – just as in the discrete model – two eavesdroppers may or may not play the same action against one another.

To analyse our eavesdropping model when we have continuous strategies, we use evolutionary invasion analysis [27], also known as adaptive dynamics [28], [29], [30]. This assumes homogeneous populations, rare mutants and small phenotypic effects from mutations. It allows us to investigate whether a mutant is able to invade a monomorphic population with a slightly different genotype and go to fixation. If mutations are small, and rare relative to the time to fixation, the genotype making up the population can move around the genotype space over time via a large number of small evolutionary steps. We thus seek the evolutionary attractors for our genotype space [28], [29], [30].

As for the discrete strategies model, we start with expressions for the probability that a player has won its last encounter as a function of the resident's genotype, and use these to construct expressions for the expected fitness of a resident and a mutant as
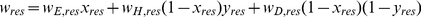
(9)and
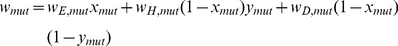
(10)respectively. The fitnesses *w_E,mut_*, *w_H,mut_*, and *w_D,mut_* are all functions of relatedness *r*, as a mutant will either interact with an individual of its own genotype (with probability *r*) or with an individual with the resident genotype (with probability 1–*r*, since the resident genotype is assumed to be at fixation when the mutant appears). The relative fitness *W* of a mutant is the difference between the mutant's fitness and the weighted average fitness of the population, i.e. *W* = *w_mut_*−(*sw_mut_*+(1−*s*)*w_res_*), where *s* is the frequency of the mutant in the population. To assess the susceptibility of genotypes to invasion by mutants, we assume that the mutant is rare (i.e. 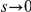), and so *W* simplifies to *W*≈*w_mut_*−*w_res_*. We can then use this to derive the selection gradients, *W_x_* and *W_y_*, and find equilibrium values of *x_i_* and *y_i_* by solving the first order conditions:
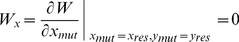
(11)and
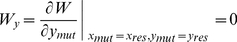
(12)for 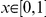 and 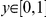, which provides the parameters for the equilibrium genotype. Material S1 provides more details of the derivation. This approach assumes a homogeneous population (where mutants are at a negligible density relative to resident individuals), that mutants with a positive invasion fitness (that is, those that do better than the resident strategy) will successfully invade and be driven to fixation, and that mutation occurs in small steps, with *x_mut_* and *y_mut_* deviating only slightly from *x_res_* and *y_res_* [28], [29], [30]. This model thus differs conceptually from the discrete strategies model, in which the population was genotypically heterogeneous at equilibrium.

Without population structure (*r* = 0), there is a single equilibrium genotype that gives rise to eavesdropper, hawk and dove phenotypes in accordance with parameters *x* ^*^ = 8*v*(*c*−*v*)/(*c* ^2^+8*cv*) and *y* ^*^ = *v*(*c*+8*v*)/(*c* ^2^+8*v* ^2^). These phenotype frequencies match the genotype frequencies of both our discrete strategies model and Johnstone's eavesdropping model [7].

Our results differ, however, when we incorporate relatedness into the population (i.e. *r*>0). As relatedness increases, the equilibrium genotype parameter *y* ^*^ falls, since avoiding escalated conflicts by playing *dove* is always more favourable when interactions with relatives become more common. If the value of the resource *v* is sufficiently high compared to the cost of fighting *c*, then the equilibrium eavesdropping frequency *x* ^*^ peaks at intermediate levels of relatedness before dropping to 0 when relatedness reaches 1 (Figure 2). Unlike the discrete strategies model, however, eavesdropping never goes to fixation. The peak frequency of eavesdropping occurs at lower relatedness the smaller is *v*/*c*; if *v*/*c* is sufficiently small then the frequency of eavesdropping decreases monotonically to 0 as *r* increases (Figure 3).

When eavesdroppers make errors in determining the outcomes of their adversaries' prior encounters, we find that the equilibrium frequency of eavesdropping peaks at a lower level of relatedness compared to error-free eavesdropping or, if *v*/*c* is small, is lower at all values of *r*. As with the discrete strategies model, we model these errors by introducing an ‘accuracy’ parameter 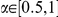, describing the probability that an eavesdropper correctly perceives an adversary's prior outcome (formal results not shown but available from the authors on request). The equilibrium frequency of eavesdroppers may actually increase with eavesdropping errors when relatedness is low and *v*/*c* high, since errors cause eavesdroppers to avoid some escalated fights against other eavesdroppers, and this is favourable. The frequencies of both individual aggression and escalated conflict are always higher in the eavesdropping model than in the model without eavesdropping (Figure 4; see also Material S1), but the frequency of escalated conflict is less than would be expected from simply squaring the frequency of individual aggression. As eavesdropping errors increase (i.e. α approaches 0.5), aggression and escalated conflict converge to the same level as observed in the model without eavesdropping, although eavesdropping still takes place in equilibrium.

## Discussion

Although players cannot distinguish kin from non-kin directly in our model, nor accurately predict what action an opponent will choose in a future encounter, eavesdropping provides scope for a conditional response such that *hawk* is played against opponents that are on average comparatively likely to play *dove*, and *vice versa*.

Our models show that eavesdropping is most successful at intermediate levels of relatedness. By contrast, at high relatedness, individuals maximise their inclusive fitness by always playing *dove*, which consequently goes to fixation. This is similar to the basic hawk-dove game, in the absence of eavesdropping, in which doves also go to fixation if relatedness is high – i.e. if *r*≥*v*/(*v*+*c*) under discrete strategies, and if *r* = 1 under continuous strategies [15]. It also echoes the classic result in the general 2×2 game where the broad pattern of a stable polymorphism at intermediate relatedness and a monomorphic equilibrium at high relatedness is associated with negatively additive payoffs [15], [31], [32]. The formal extension of that result to our models may be useful in placing eavesdropping in the context of more general 3×3 games, but is complicated by the presence of three actions rather than two, and the fact that payoffs are themselves a function of population frequencies (via the probability of winning a previous encounter).

In our models, eavesdropping yields no useful information at high relatedness, since eavesdroppers essentially face a uniform population of adversaries whose members are otherwise interacting only with each other: eavesdropping relatives in the discrete strategies case, or doves in the continuous strategies case. Consequently, rare eavesdroppers play *hawk* and *dove* with equal probability but their choice in any encounter is uncorrelated with their opponent's choice in that encounter. They fare strictly worse than doves under discrete strategies because they sometimes bear the cost of fighting in escalated conflicts, and are unable to increase in frequency under continuous strategies for the same reason. At low relatedness, negative frequency dependent selection means that neither eavesdroppers, hawks nor doves can go to fixation, consistent with Johnstone [7] in which relatedness is zero. A resident population of hawks can be invaded by either doves or eavesdroppers; doves can be invaded by either eavesdroppers or hawks; and eavesdroppers can also be invaded by hawks (and by doves, but only when the relative cost of fighting is high, specifically *v/c*≤4).

Interacting with relatives relaxes the frequency dependence that maintains all three strategies in equilibrium with zero relatedness. Although this result is borne out qualitatively in both the discrete- and continuous strategies models, the equilibria of the two models differ when relatedness is positive. For example, the discrete strategies model allows eavesdroppers or doves to go to fixation given suitable model parameters; under the continuous strategies model eavesdroppers never go to fixation, and doves only become fixed at *r* = 1. This contrasts with the case where there is no relatedness (i.e. *r* = 0), in which the genotype frequencies under discrete strategies are the same as the respective probabilities under continuous strategies, and eavesdropping never goes to fixation [7]. This divergence between discrete and continuous models in structured populations is also a known feature of the hawk-dove game without eavesdropping [15].

The equilibrium frequency of eavesdropping may be regarded as a measure of the value of eavesdropping for a given set of model parameters. This differs from the usual measure of the value of eavesdropping, which is the selection gradient given model parameters and genotype frequencies – that is, the fitness of an eavesdropping player relative to hawks or doves under discrete strategies or, in the case of continuous strategies, the change in genotype fitness from a small increase in the proportion of eavesdropping progeny. The selection gradient varies with the frequencies of eavesdroppers, hawks and doves, and also with *v*/*c* and *r*. When the gradient is positive, selection favours an increase in the frequency of eavesdropping which tends to erode the value of eavesdropping, since eavesdropping opponents are comparatively unpredictable [7]. However, the selection gradient will be zero at any equilibrium where eavesdroppers attain frequency strictly between zero and one. It is therefore useful for examining the evolutionary dynamics of eavesdropping, but less helpful for comparing the adaptive value of eavesdropping between different biological settings as captured by parameters *v*/*c* and *r* in our models. The equilibrium frequency of eavesdroppers, on the other hand, is useful for this purpose.

The value of eavesdropping as measured by the selection gradient is closely related to the value of information in our model [33], [34]. The value of eavesdropping is the benefit of observing a simple social cue and responding in a specified way – i.e. play *hawk* (*dove*) against perceived losers (winners) – which may be positive or negative. In contrast, the value of information is the net fitness benefit from responding *optimally* once observations have reduced prior uncertainty, and is always non-negative [35]. The value of eavesdropping is non-negative and equivalent to the value of information if the outcome of fights is a sufficiently reliable predictor of opponents' future actions that it is optimal to play *hawk* (dove) against perceived losers (winners). But if the outcomes of fights are sufficiently misinformative about the likelihood of an opponent playing *hawk* or *dove* in subsequent fights, the optimal response to the social cue may be either to play *hawk* or to play *dove* unconditionally – in other words, to ignore the social cue. In this case, the value of information is zero, since receiving the cue changes neither choice of action nor outcome. But the value of eavesdropping is negative, since the response conditioned on the social cue yields lower fitness than the best unconditional response that could be employed without such cue. The value of eavesdropping may be negative (and the value of information zero) when cues fail to reduce prior uncertainty sufficiently (are too uninformative) about whether a current opponent won or lost in a previous round, and/or when knowing this fails to improve payoffs from current and future bouts.

The value of eavesdropping (and, correspondingly, the value of information), involves a number of components. Firstly, an immediate direct fitness effect from altering the player's payoff in the current round, by enabling the player to distinguish (albeit imperfectly) opponents who are more likely to play *hawk* from those who are more likely to play *dove*. The size of this effect is determined by the frequencies of eavesdroppers, hawks and doves, the relative payoffs (influencing the value of any available information) which are functions of *v*/*c*, as well as the probabilities that each type won its last encounter, since these affect the average ability of an eavesdropping player to predict whether an opponent will play *hawk* or *dove* (the availability of information). Secondly, a fitness effect arising from a mutant effectively facing a different population of opponents than a resident (impacting both value and availability of information). Thirdly, an accumulation of effects in future rounds because an increased probability of winning this round also implies an increased probability of winning against an eavesdropper in the next round, since an eavesdropper plays *dove* if it perceives that its opponent won its last encounter. The net result of all these effects is captured in the relative fitness functions for the different genotypes.

Our models highlight the potential for relatedness to enhance selection for eavesdropping. The relatedness parameter *r* describes the probability of interacting with a similar partner in a given interaction, relative to chance. While we have not specified how such relatedness arises, the mechanisms invoked in the kin selection literature usually involve either kin recognition or limited dispersal [12], [22], [36]. Our *r* is best interpreted as arising from limited dispersal, since we only model interactions with a single level of relatedness. Many species face dispersal limitations, which may help suppress conflict by increasing the relatedness of opponents; however, this effect may be negated to the extent that relatives also compete to reproduce or for other resources [37], [38], [39]. We have chosen to keep our model simple, and thus assume that any resource competition is relatively global (i.e. non-dispersal limited) compared to the conflict stage captured by our game. Such global competition, with local social interactions, would likely be found in, for example, interactions between nestmates or between young raised on a territory, prior to dispersal and competition for mates or territories [39].

Our models, in which direct assessment of relatedness or strategy is unavailable, predict that eavesdropping will be most favoured at intermediate levels of population structure. More generally, our results highlight the importance of explicitly considering genetic relatedness in addition to the nature and extent of social interactions when exploring the evolution of cognitive abilities. (While more demanding behaviours can easily be found – for example among corvids and primates – eavesdropping likely represents a significant cognitive challenge, at a minimum requiring recognition of individuals and the capacity to process and remember past observations of those individuals.) We suggest that our predictions can be tested directly. Earley and Dugatkin [9] have already demonstrated eavesdropping in the green swordtail *Xiphophorus helleri*. One test of our model would be to repeat the same experimental protocol with *X. helleri* but to vary the degree of relatedness among each trio of lab-raised fish. An alternative test would be to select several *Xiphophorus* species that exhibit different degrees of population structure and to repeat the same experimental protocol across those species. More indirect evidence might come from examining brain size or cognitive capacity as a function of population structure for each species, since some authors have argued that larger brains evolved in part to process the demands of living in a highly social environment [40], [41], [42].

## Supporting Information

Material S1 **Provides additional details of the solutions to the models and of the results.**
(PDF)
