# Supplementary material for: Kin Selection and the Evolution of Social Information Use in Animal Conflict
Source: PLoS One. 2012 Feb 22;7(2):e31664. doi: 10.1371/journal.pone.0031664 (PMC3285175; doi:10.1371/journal.pone.0031664)
Supplement: Material S1 — Provides additional details of the solutions to the models and of the results. (PDF) [file pone.0031664.s001.pdf]

KIN SELECTION AND THE EVOLUTION OF INFORMATION USE IN ANIMAL CONFLICT

SUPPLEMENTARY MATERIAL S1

CHRISTOPHER C.M. BAKER, SASHA R.X. DALL AND DANIEL J. RANKIN

1. Solution to the discrete strategies model

In the discrete strategies model, there are three 'genotypes' – E, H and D – each corresponding to a different strategy that may be thought of as a phenotype. An individual with the hawk genotype always plays the action *hawk*; a dove always plays *dove*; and an eavesdropper plays the conditional eavesdropping strategy, which may dictate either *hawk* or *dove* in any given encounter. We wish to determine the frequencies of the three genotypes in equilibrium.

Let f_E, f_H and f_D denote the frequencies of genotypes E, H and D respectively. These frequencies change from generation to generation, but not from interaction to interaction within an individual's lifetime. Let $p_{E,t}$, $p_{H,t}$ and $p_{D,t}$ denote the probability that an individual of type E, H and D respectively wins its encounter at time t. The relatedness parameter r represents population structure in the model, and measures the probability that a player's opponent has the same genotype, relative to the probability for a randomly drawn member of the population. With probability r, a player of type $i \in \{\mathrm{E}, \mathrm{H}, \mathrm{D}\}$ interacts with a type i opponent. In addition, with probability $(1-r)$ the player interacts with a randomly chosen individual. Thus, a player of type i plays another type i individual with probability $r + (1-r)f_i$, and plays an opponent of type $j \neq i$ with probability $(1-r)f_j$. Thus, when $r = 1$, pairs of players always have the same genotype; when $r = 0$, players interact with each genotype in proportion to the population frequencies. Although relatedness may be negative, we restrict our analysis to the more traditional case of $r \in [0, 1]$. Note that r measures assortment at the level of the genotype rather than phenotype or action: an eavesdropper meets another eavesdropper with probability $r+(1-r)f_E$, but in a given interaction the two may or may not play the same action, since each player's action depends on the outcome of its opponent's previous encounter.

The probability that a type i player wins its encounter at time $t+1$ depends on whether it won at time t.

$$\begin{aligned}
p_{E,t+1} &= r\left(\frac{p_{E,t}^2}{2} + \frac{(1-p_{E,t})^2}{2} + p_{E,t}(1-p_{E,t})\right) \\
&\quad +(1-r)\left(f_E\left(\frac{p_{E,t}^2}{2} + \frac{(1-p_{E,t})^2}{2} + p_{E,t}(1-p_{E,t})\right)\right. \\
&\qquad \left. +f_H\frac{(1-p_{H,t})}{2} + f_D\left(1-\frac{p_{D,t}}{2}\right)\right) \qquad (1.1) \\
p_{H,t+1} &= \frac{r}{2} + (1-r)\left(f_E\left(\frac{1-p_{H,t}}{2} + p_{H,t}\right) + \frac{f_H}{2} + f_D\right) \\
p_{D,t+1} &= \frac{r}{2} + (1-r)\left(f_E\frac{p_{D,t}}{2} + \frac{f_D}{2}\right)
\end{aligned}$$

These equations, and those that follow, assume no eavesdropping errors. Errors can be introduced through a parameter $\alpha \in (0.5, 1]$ denoting the probability that an eavesdropper's observation is accurate, i.e. the eavesdropper perceives a win when the true outcome was a win, and perceives a loss when the true outcome was a loss; with probability $(1-\alpha)$, the eavesdropper perceives a win when the true outcome was a loss, or *vice versa*. The eavesdropper plays *dove* in any interaction where the opponent's prior encounter was perceived as a

win, and otherwise plays *hawk*. Thus, the probability that an eavesdropper plays *hawk* against an opponent of type i at time $t+1$ is $\alpha(1-p_{i,t})+(1-\alpha)p_{i,t}$, while the probability that it plays *dove* is $\alpha p_{i,t}+(1-\alpha)(1-p_{i,t})$. For brevity, we only show results for $\alpha=1$, but finding results for $\alpha<1$ simply involves substituting these expressions for the probabilities in the solution presented here.

We solve for the steady state probabilities by setting $p_{i,t+1}=p_{i,t}$ for $i\in\{\mathrm{E},\mathrm{H},\mathrm{D}\}$ which, dropping the time subscript, gives us

$$\begin{aligned} p_E &= \frac{1+r+2f_D(1-r)}{2(2-f_E(1-r))} \\ p_H &= \frac{1+f_D(1-r)}{2-f_E(1-r)} \\ p_D &= \frac{r+f_D(1-r)}{2-f_E(1-r)}. \end{aligned} \tag{1.2}$$

Type i's average lifetime fitness w_i is given by the average payoff across a large number of encounters.

$$\begin{aligned} w_E &= r\left(\frac{(1-p_E)^2(v-c)}{2}+\frac{p_E^2 v}{2}+p_E(1-p_E)v\right) \\ &\quad+(1-r)\left(f_E\left(\frac{(1-p_E)^2(v-c)}{2}+\frac{p_E^2 v}{2}+p_E(1-p_E)v\right)\right. \\ &\qquad\left.+f_H\frac{(1-p_H)(v-c)}{2}+f_D\left((1-p_D)v+\frac{p_D v}{2}\right)\right) \\ w_H &= r\frac{(v-c)}{2}+(1-r)\left(f_E\left(\frac{(1-p_H)(v-c)}{2}+p_H v\right)+f_H\frac{(v-c)}{2}+f_D v\right) \\ w_D &= r\frac{v}{2}+(1-r)\left(f_E\frac{p_D v}{2}+f_D\frac{v}{2}\right) \end{aligned} \tag{1.3}$$

From generation to generation, types E, H and D reproduce proportional to their average fitnesses according to standard replicator dynamics. In equilibrium with all three types present, it must be the case that $w_E=w_H=w_D$. Substituting (1.2) into (1.3) and solving for the frequencies f_E, f_H and f_D gives

$$\begin{aligned} f_E &= \frac{4cv\left(r^2+r+2\right)+4v^2\left(r^2+r-2\right)-c^2r(1-r)}{c(1-r)(8v+c(1-r))} \\ f_H &= \frac{v}{c}-\frac{r}{1-r} \\ f_D &= \frac{(c(1+r)-v(1-r))(c(1-r)-4rv)}{c(1-r)(8v+c(1-r))}. \end{aligned} \tag{1.4}$$

These frequencies sum to 1 and represent the interior equilibrium so long as f_E, f_H and f_D are each strictly between 0 and 1, which is the case when

$$r<min\{\frac{c}{4v+c},\frac{v}{v+c}\}. \tag{1.5}$$

The first of the terms in (1.5) binds when $v/c\geq 0.5$, while the second binds when $v/c\leq 0.5$. When (1.5) is not satisfied, the equilibrium must either be monomorphic or dimorphic. Equilibrium frequencies for dimorphic populations can be calculated by setting the relevant pair of fitnesses from (1.3) equal to each other, substituting from (1.2) and solving. Again the calculated frequencies must lie between 0 and 1, and the third genotype must not be able to invade.

For $v/c>0.5$, Es and Hs coexist stably when

$$\frac{c}{4v+c}\leq r<\frac{4v+c}{7c+4v}, \tag{1.6}$$

with Es at frequency

$$f_E = \frac{1}{8c(1-r)^2}\Bigg(c(5r^2-6r+13)-4v(1-r) - \sqrt{c^2(9r^4+52r^3+54r^2-12r+41)+16v^2-8cv(5r^2+10r-3)/(1-r)}\Bigg) \tag{1.7}$$

and $f_H = 1 - f_E$ and $f_D = 0$.

Es go to fixation when $v/c > 0.5$ and

$$\frac{4v+c}{7c+4v} \leq r < \frac{4v-c}{4v+c}. \tag{1.8}$$

When r exceeds the upper bound in (1.8) and with $v/c > 0.5$, Es and Ds can coexist, with Es at frequency

$$f_E = \frac{8v(1-r) - cr(1+r)^2}{(1-r)\left(4v(1-r)+c(1+r)^2\right)}, \tag{1.9}$$

and $f_H = 0$ and $f_D = 1 - f_E$, so long as the value of f_E given by the numerator of (1.9) is positive (since the denominator is always positive). Ds go to fixation for sufficiently high r, when the numerator of (1.9) falls below zero.

For $v/c < 0.5$, Es and Hs do not stably coexist for any r, nor do Es go to fixation. Es and Ds coexist with the frequency of Es given by (1.9) when $r > \frac{v}{v+c}$, again so long as the value of f_E given by (1.9) is positive. As for $v/c > 0.5$, Ds go to fixation for sufficiently high r, when the numerator of (1.9) is below zero.

2. Results for the discrete strategies model

Equilibrium frequencies. When relatedness is low, all three genotypes coexist in equilibrium. Hawks are comparatively common when the value of the resource is high compared to the cost of fighting (i.e. $\frac{v}{c}$ close to 1). Doves are common when the value of the resource is low, and eavesdroppers peak in frequency for intermediate resource values, as per Johnstone (2001). Doves go to fixation when relatedness is high enough in our model. The level of relatedness required for doves to go to fixation increases with the relative resource value. Eavesdroppers peak in frequency at intermediate relatedness, and go to fixation when the value of the resource is also sufficiently high. Figure 2.1 summarises the equilibrium genotype frequencies over $\{r, \frac{v}{c}\}$ space.

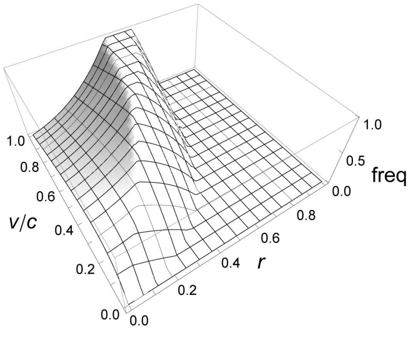

(A) Eavesdroppers

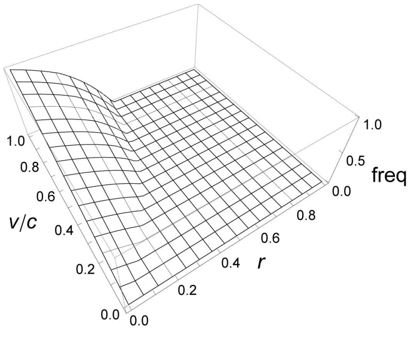

(B) Hawks

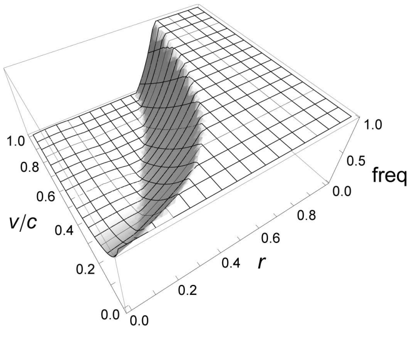

(C) Doves

Figure 2.1. Equilibrium frequencies for the discrete strategies model

Individual aggression. The frequency of individual aggression is the average proportion of players who play *hawk* in any given encounter. In the hawk-dove game without eavesdropping, i.e. the discrete strategies model of Grafen (1979), this is simply the frequency of hawks in the population, calculated according to Grafen's equation 6. In the game with eavesdropping, this still includes all hawks, but also any eavesdroppers who face an opponent that lost the last encounter:

$$f_H + f_E \left(r(1 - p_E) + (1 - r) \left(f_E(1 - p_E) + f_H(1 - p_H) + f_D(1 - p_D) \right) \right). \tag{2.1}$$

In the games both with and without eavesdropping, the frequency of individual aggression approaches 1 when relatedness r is zero and the relative value of the resource $\frac{v}{c}$ approaches 1. Individual aggression drops as relatedness increases and as the relative value of the resource falls (Figures 2.2a and b). In both cases, individual aggression is 0 when doves go to fixation in equilibrium. In the eavesdropping model, this occurs when the numerator of (1.9) is negative. In the model without eavesdropping, this occurs when $r > \frac{v}{v+c}$. For any given $\frac{v}{c}$, doves go to fixation at lower r in Grafen's (1979) model than in the model with eavesdropping. The frequency of individual aggression is higher with eavesdropping than without, as long as r is not so high that doves go to fixation in both models, in which case there is no difference (Figure 2.2c).

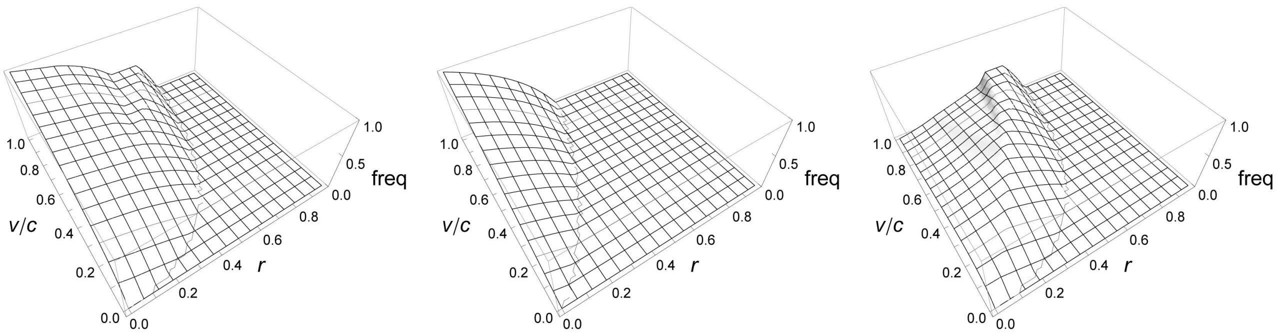

(A) With eavesdropping (B) Without eavesdropping (C) Difference

FIGURE 2.2. Level of individual aggression in the discrete strategies hawk-dove game (a) with and (b) without eavesdropping. The frequency of individual aggression is the expected proportion of *hawk* actions in equilibrium. (c) shows the difference in the frequency of individual aggression between the two models, i.e. with eavesdropping minus without eavesdropping.

Escalated aggression. The frequency of escalated aggression is the proportion of encounters in which both players play *hawk*. In our model, a reasonable 'naïve' estimate of the frequency of escalated conflict given the level of individual aggression might be the square of (2.1). This is the level of escalated conflict that would obtain if all players simply chose *hawk* with the probability given by (2.1). The actual frequency of escalated conflict differs from this estimate for two reasons: firstly, because the probability that an eavesdropper plays *hawk* is not generally independent of the probability that its opponent also plays *hawk*; secondly, because population structure means that players are more likely to interact with their own type. The actual expected level of escalated conflict is

$$f_E(r(1 - p_E)^2 + (1 - r)(f_E(1 - p_E)^2 + f_H(1 - p_H))) + f_H(r + (1 - r)(f_E(1 - p_H) + f_H)). \tag{2.2}$$

Similar to individual aggression, the frequency of escalated conflicts approaches 1 when relatedness r is zero and the relative value of the resource $\frac{v}{c}$ approaches 1. Escalated aggression drops as relatedness increases and as the relative value of the resource falls (Figures 2.3a and b). In both cases, escalated conflicts never occur when doves go to fixation in equilibrium. The frequency of escalated conflicts is higher with eavesdropping than without, as long as r is not so high that doves go to fixation in both models, in which case there is no difference (Figure 2.3c).

The higher frequency of escalated conflict in the eavesdropping model is intuitively consistent with the higher level of individual aggression. This in turn reflects the additional value of winning an encounter, as described by

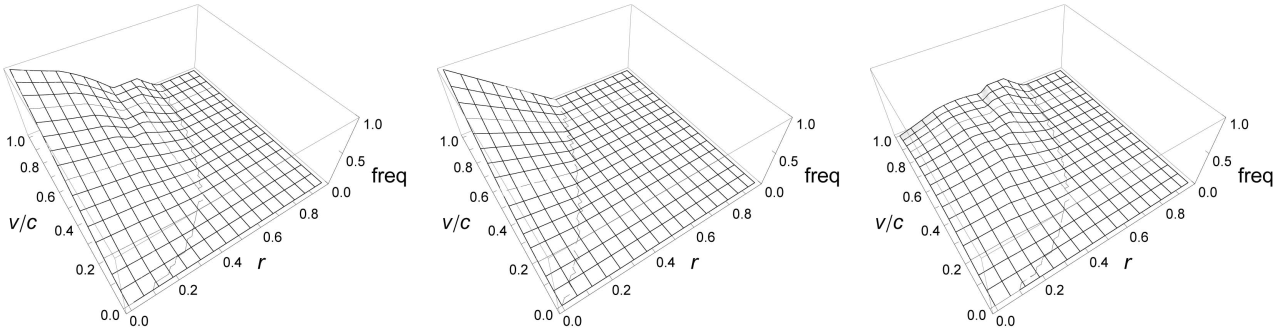

(A) With eavesdropping (B) Without eavesdropping (C) Difference

FIGURE 2.3. Level of escalated aggression in the discrete strategies hawk-dove game (a) with and (b) without eavesdropping. The frequency of escalated aggression is the expected proportion of *hawk-hawk* encounters in equilibrium. (c) shows the difference in the frequency of escalated aggression between the two models, i.e. with eavesdropping minus without eavesdropping.

Johnston (2001) – in addition to the payoff from the current encounter, winning also increases the probability of winning against eavesdroppers in future rounds. However, the frequency of escalated conflict given by (2.2) is not generally equal to the square of (2.1) – this is only the case when the population is monomorphic in equilibrium. Johnstone (2001) notes that the frequency of escalated conflict is lower than the square of individual aggression when relatedness is zero. With positive relatedness, however, the frequency of escalated conflict may be either higher or lower than the square of individual aggression. It tends to exceed the square of individual aggression when r is high, as long as r is not so high that doves go to fixation in both models (Figure 2.4).

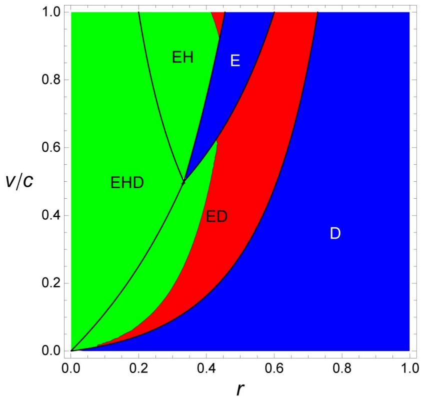

FIGURE 2.4. Level of escalated aggression in the eavesdropping game relative to the square of individual aggression. The frequency of escalated aggression is the expected proportion of *hawk-hawk* encounters in equilibrium. The green (red) denotes combinations of $\frac{v}{c}$ and r for which the frequency of escalated aggression is lower (higher). The blue shows combinations for which the two are the same. For reference, the equilibria described in the results and shown in Figure 1 of the main paper are also marked.

3. Solution to the continuous strategies model

In the continuous strategies model, a 'genotype' i displays phenotypic plasticity as described by two parameters, x_i and y_i. Of the individuals with genotype i, a proportion x_i take on an eavesdropper phenotype throughout their lives and thus play the conditional eavesdropping strategy in every interaction. A proportion $(1 - x_i)y_i$ take on the hawk phenotype and thus always play the action *hawk*; likewise a proportion $(1-x_i)(1-y_i)$ take on the dove phenotype and so always play the action *dove*. Since the relevant evolutionary dynamics take place in this model at the genotype level, the fitness of genotype i is the weighted average fitness of its three phenotypes,

where phenotype fitness is again measured as the average across all interactions during an individual's lifetime. We wish to determine which genotypes at fixation cannot be invaded by phenotypically-nearby mutants.

As in the discrete strategies model described in section 1, we allow for non-random assortment among genotypes by permitting non-zero relatedness. The exogenous relatedness parameter r measures the probability that a player's opponent has the same genotype, relative to the probability for a randomly drawn member of the population. An individual with genotype i plays another type i individual with probability $r + (1-r)f_i$, and plays an opponent of type $j \neq i$ with probability $(1-r)f_j$, where the $f_.$ are the frequencies of the genotypes in the population. We again restrict attention to $r \in [0,1]$. Note that assortment is also measured at the genotype level, and when two players have the same genotype this means that they share the same values for x and y. However, at the time of the interaction, their phenotypes are already determined as eavesdropper, hawk or dove, and the two players may or may not share the same phenotype. Additionally – just as in the discrete model – two eavesdroppers may or may not play the same action against one another.

Now, consider firstly a resident genotype at fixation. Since the population is large, the frequency of the resident genotype is essentially 1 and the frequency of any other genotype is essentially 0, even if there are mutants. Consequently, $r + (1-r)f_i \approx 1$ for the resident genotype i and $(1-r)f_j \approx 0$ for any other genotype $j \neq i$, so the relatedness parameter is not apparent in the equation for the resident's fitness. With a resident genotype at fixation, the fitness of a representative individual is a function of the phenotype frequencies for the resident. Recall that a genotype gives rise to players with phenotypes E, H and D in proportions determined by its parameters x and y. The frequency of Es among residents is given by x_{res}, while Hs arise with frequency $(1-x_{res})y_{res}$ and Ds with frequency $(1-x_{res})(1-y_{res})$. Let $p_{i,res,t}$ denote the probability that a resident of type $i \in \{\mathrm{E},\mathrm{H},\mathrm{D}\}$ wins its encounter at time t. Now, the probability that it wins its encounter at time $t+1$ is given by

$$\begin{aligned}
p_{E,res,t+1} &= \left(\frac{p^2_{E,res,t}}{2} + \frac{1}{2}(1-p_{E,res,t})^2 + p_{E,res,t}(1-p_{E,res,t})\right)x_{res} \\
&\quad + \frac{1}{2}(1-p_{H,res,t})(1-x_{res})y_{res} + \left(1 - \frac{p_{D,res,t}}{2}\right)(1-x_{res})(1-y_{res}) \\
p_{H,res,t+1} &= \left(\frac{1-p_{H,res,t}}{2} + p_{H,res,t}\right)x_{res} + \frac{1}{2}(1-x_{res})y_{res} + (1-x_{res})(1-y_{res}) \qquad (3.1) \\
p_{D,res,t+1} &= \frac{p_{D,res,t}}{2}x_{res} + \frac{1}{2}(1-x_{res})(1-y_{res})
\end{aligned}$$

These equations, and those that follow, assume no eavesdropping errors. Errors can be introduced through a parameter $\alpha \in (0.5, 1]$ denoting the probability that an eavesdropper's observation is accurate i.e. the eavesdropper perceives a win when the true outcome was a win, and perceives a loss when the true outcome was a loss; with probability $(1-\alpha)$, the eavesdropper perceives a win when the true outcome was a loss, or *vice versa*. The eavesdropper plays *dove* in any interaction where the opponent's prior encounter was perceived as a win, and otherwise plays *hawk*. Thus, the probability that an eavesdropper plays *hawk* against an opponent of type i at time $t+1$ is $\alpha(1-p_{i,t}) + (1-\alpha)p_{i,t}$, while the probability that it plays *dove* is $\alpha p_{i,t} + (1-\alpha)(1-p_{i,t})$. For brevity, we only show results for $\alpha = 1$, but finding results for $\alpha < 1$ simply involves substituting these expressions for the probabilities in the solution presented here.

We solve for the equilibrium probabilities by setting $p_{i,res,t+1} = p_{i,res,t}$ for $i \in \{\mathrm{E},\mathrm{H},\mathrm{D}\}$ which, dropping the time subscript, gives us

$$\begin{aligned}
p_{E,res} &= \frac{\frac{1}{2} + (1-x_{res})(1-y_{res})}{2-x_{res}} \\
p_{H,res} &= \frac{1 + (1-x_{res})(1-y_{res})}{2-x_{res}} \qquad (3.2) \\
p_{D,res} &= \frac{(1-x_{res})(1-y_{res})}{2-x_{res}}
\end{aligned}$$

A mutant genotype gives rise to E, H and D in slightly different proportions to the resident, but also effectively faces a different set of opponents due to the effect of population structure. If the resident genotype i is at fixation, then f_i is essentially 1 and $f_{j\neq i}$ is essentially 0. Thus, the mutant genotype j encounters another mutant with frequency r, and encounters the resident with frequency $(1-r)$. Now, if we denote the probability that a mutant of type $i \in \{\mathrm{E},\mathrm{H},\mathrm{D}\}$ wins its encounter at time t as $p_{i,mut,t}$, then the probability that it wins its encounter at time $t+1$ is given by

$$\begin{aligned}
p_{E,mut,t+1} &= r\Bigg(\left(\frac{p_{E,mut,t}^2}{2}+\frac{1}{2}(1-p_{E,mut,t})^2+p_{E,mut,t}(1-p_{E,mut,t})\right)x_{mut} \\
&\quad +\frac{1}{2}(1-p_{H,mut,t})(1-x_{mut})y_{mut}+\left(1-\frac{p_{D,mut,t}}{2}\right)(1-x_{mut})(1-y_{mut})\Bigg) \\
&\quad +(1-r)\Bigg(\left(\frac{1}{2}(1-p_{E,res,t})(1-p_{E,mut,t})+(1-p_{E,res,t})p_{E,mut,t}+\frac{p_{E,res,t}p_{E,mut,t}}{2}\right)x_{res} \\
&\quad +\frac{1}{2}(1-p_{H,res,t})(1-x_{res})y_{res}+\left(1-\frac{p_{D,res,t}}{2}\right)(1-x_{res})(1-y_{res})\Bigg) \\
p_{H,mut,t+1} &= r\left(\left(\frac{1-p_{H,mut,t}}{2}+p_{H,mut,t}\right)x_{mut}+\frac{1}{2}(1-x_{mut})y_{mut}+(1-x_{mut})(1-y_{mut})\right) \\
&\quad +(1-r)\left(\left(\frac{1-p_{H,mut,t}}{2}+p_{H,mut,t}\right)x_{res}+\frac{1}{2}(1-x_{res})y_{res}+(1-x_{res})(1-y_{res})\right) \\
p_{D,mut,t+1} &= r\left(\frac{p_{D,mut,t}}{2}x_{mut}+\frac{1}{2}(1-x_{mut})(1-y_{mut})\right) \\
&\quad +(1-r)\left(\frac{p_{D,mut,t}}{2}x_{res}+\frac{1}{2}(1-x_{res})(1-y_{res})\right) \qquad (3.3)
\end{aligned}$$

Using steady state values for the $p_{i,res,t+1}$ from (3.2), we can set $p_{i,mut,t+1} = p_{i,mut,t}$ for $i \in \{\mathrm{E},\mathrm{H},\mathrm{D}\}$ and solve for the steady state probabilities for the mutant. Omitting the time subscripts, we have

$$\begin{aligned}
p_{E,mut} &= \frac{1}{2(2-(1-r)x_{res})(2-rx_{mut}-(1-r)x_{res})}\Big(2(1-r)^2x_{res}^2(1-y_{res}) \\
&\quad -(1-r)x_{res}(r(2x_{mut}y_{mut}-2y_{mut}-2x_{mut}-1)-2(3-2r)y_{res}+7) \\
&\quad +r(2(3-r)(x_{mut}y_{mut}+y_{res}-y_{mut})-(5-r)x_{mut})-4y_{res}+6\Big) \\
p_{H,mut} &= 1-\frac{r(1-x_{mut})y_{mut}+(1-r)(1-x_{res})}{2-rx_{mut}-(1-r)x_{res}} \\
p_{D,mut} &= \frac{1+r(x_{mut}y_{mut}-x_{mut}-y_{mut})+(1-r)(x_{res}y_{res}-x_{res}-y_{res})}{2-rx_{mut}-(1-r)x_{res}}
\end{aligned}$$

Now, the average payoff for residents is given by

$$w_{res} = w_{E,res}x_{res}+w_{H,res}(1-x_{res})y_{res}+w_{D,res}(1-x_{res})(1-y_{res}) \qquad (3.4)$$

where

$$\begin{aligned}
w_{E,res} &= \left(\frac{v-c}{2}(1-p_{E,res})^2+v(1-p_{E,res})p_{E,res}+\frac{v}{2}p_{E,res}^2\right)x_{res} \\
&\quad +\frac{v-c}{2}(1-p_{H,res})(1-x_{res})y_{res}+\left(v(1-p_{D,res})+\frac{v}{2}p_{D,res}\right)(1-x_{res})(1-y_{res}) \\
w_{H,res} &= \left(\frac{v-c}{2}(1-p_{H,res})+vp_{H,res}\right)x_{res}+\frac{v-c}{2}(1-x_{res})y_{res}+v(1-x_{res})(1-y_{res}) \\
w_{D,res} &= \frac{v}{2}p_{D,res}x_{res}+\frac{v}{2}(1-x_{res})(1-y_{res}) \qquad (3.5)
\end{aligned}$$

and for mutants it is

$$w_{mut} = w_{E,mut}x_{mut} + w_{H,mut}(1-x_{mut})y_{mut} + w_{D,mut}(1-x_{mut})(1-y_{mut}) \tag{3.6}$$

where

$$\begin{aligned}
w_{E,mut} &= r\Bigg(\left(\frac{v-c}{2}(1-p_{E,mut})^2 + \frac{v}{2}p_{E,mut}^2 + vp_{E,mut}(1-p_{E,mut})\right) x_{mut} \\
&\qquad + \frac{v-c}{2}(1-p_{H,mut})(1-x_{mut})y_{mut} + \left(v(1-p_{D,mut}) + \frac{v}{2}p_{D,mut}\right)(1-x_{mut})(1-y_{mut})\Bigg) \\
&\quad +(1-r)\Bigg(\left(\frac{v-c}{2}(1-p_{E,res})(1-p_{E,mut}) + v(1-p_{E,res})p_{E,mut} + \frac{v}{2}p_{E,res}p_{E,mut}\right) x_{res} \\
&\qquad + \frac{v-c}{2}(1-p_{H,res})(1-x_{res})y_{res} + \left(v(1-p_{D,res}) + \frac{v}{2}p_{D,res}\right)(1-x_{res})(1-y_{res})\Bigg) \\
w_{H,mut} &= r\Bigg(\left(\frac{v-c}{2}(1-p_{H,mut}) + vp_{H,mut}\right) x_{mut} + \frac{v-c}{2}(1-x_{mut})y_{mut} + v(1-x_{mut})(1-y_{mut})\Bigg) \\
&\quad +(1-r)\Bigg(\left(\frac{v-c}{2}(1-p_{H,mut}) + vp_{H,mut}\right) x_{res} + \frac{v-c}{2}(1-x_{res})y_{res} + v(1-x_{res})(1-y_{res})\Bigg) \\
w_{D,mut} &= r\left(\frac{v}{2}p_{D,mut}x_{mut} + \frac{v}{2}(1-x_{mut})(1-y_{mut})\right) + (1-r)\left(\frac{v}{2}p_{D,mut}x_{res} + \frac{v}{2}(1-x_{res})(1-y_{res})\right)
\end{aligned} \tag{3.7}$$

Now, the relative fitness of a mutant is

$$W = w_{mut} - w_{res}. \tag{3.8}$$

At any equilibrium with $x \in (0,1)$ and $y \in (0,1)$ we require the fitness gradients for the two parameters to be zero:

$$W_x = \left.\frac{\partial W}{\partial x_{mut}}\right|_{x_{mut}=x_{res}, y_{mut}=y_{res}} = 0 \tag{3.9}$$

and

$$W_y = \left.\frac{\partial W}{\partial y_{mut}}\right|_{x_{mut}=x_{res}, y_{mut}=y_{res}} = 0. \tag{3.10}$$

To find the equilibrium parameters (x^*, y^*) we solve (3.9) and (3.10) numerically, making use of (3.2), (3.4), (3.4) and (3.6).

4. Results for the continuous strategies model

Equilibrium frequencies. In the continuous strategies model, an equilibrium consists of a single genotype (x^*, y^*) that gives rise to eavesdropper, hawk and dove phenotypes with probabilities x^*, $(1-x^*)y^*$ and $(1-x^*)(1-y^*)$ respectively. All three phenotypes are present at positive frequency for $r \in [0,1)$ and $\frac{v}{c} \in (0,1)$. Only doves are present when $r = 1$ (and $\frac{v}{c}$ is in $(0,1)$ by assumption). This result differs from the discrete strategies model, in which eavesdroppers or doves can go to fixation with relatedness much less than 1.

As for the discrete strategies model, when the relative resource value $\frac{v}{c}$ is close to 1, the frequency of eavesdroppers peaks at intermediate levels of relatedness. As $\frac{v}{c}$ falls, eavesdropping peaks at lower levels of r. When $\frac{v}{c}$ is sufficiently low, the maximum frequency of eavesdropping occurs at $r = 0$ and, for any lower level of $\frac{v}{c}$, eavesdropping is strictly declining in relatedness (Figure 4.1a). Hawks are comparatively common when both $\frac{v}{c}$ is high and relatedness low (Figure 4.1c). Doves are common when the value of the resource is low or when relatedness is high (Figure 4.1d).

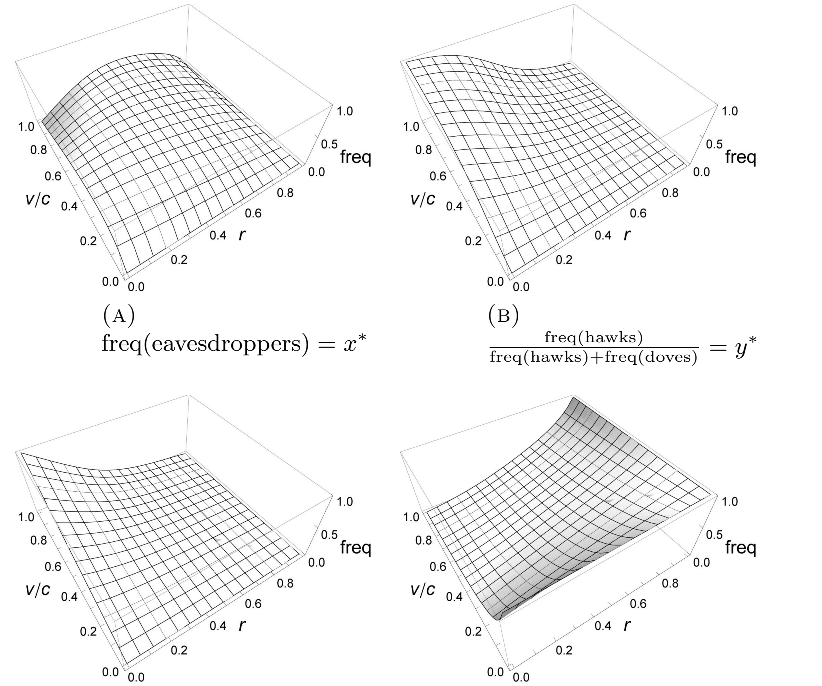

(A) freq(eavesdroppers) $= x^*$

(B) $\frac{\text{freq(hawks)}}{\text{freq(hawks)+freq(doves)}} = y^*$

(C) freq(hawks) $= (1 - x^*)y^*$

(D) freq(doves) $= (1 - x^*)(1 - y^*)$

Figure 4.1. Equilibrium frequencies for the continuous strategies model

Individual aggression. The frequency of individual aggression is the average proportion of players who play *hawk* in any given encounter. In the hawk-dove game without eavesdropping, i.e. the continuous strategies model of Grafen (1979), this is simply the probability that a player plays *hawk*, calculated according to Grafen's equation (9). In our game with eavesdropping, it includes all players with a hawk phenotype, plus any with an eavesdropper phenotype who face an opponent that lost the last encounter:

$$(1 - x^*)y^* + x^*\big(x^*(1 - p_E(x^*, y^*)) + (1 - x^*)y^*(1 - p_H(x^*, y^*)) + (1 - x^*)(1 - y^*)(1 - p_D(x^*, y^*))\big) \qquad (4.1)$$

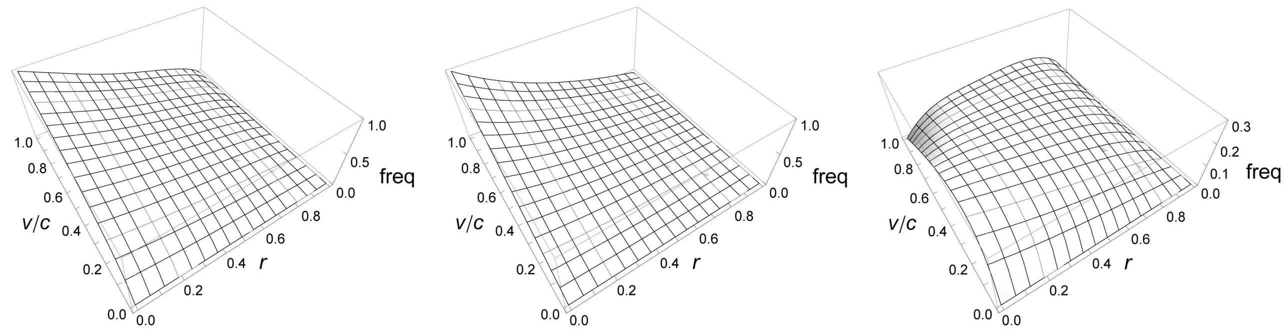

(A) With eavesdropping (B) Without eavesdropping (C) Difference

Figure 4.2. Level of individual aggression in the continuous strategies hawk-dove game (a) with and (b) without eavesdropping. The frequency of individual aggression is the expected proportion of *hawk* actions in equilibrium. (c) shows the difference in the frequency of individual aggression between the two models, i.e. with eavesdropping minus without eavesdropping.

In the games both with and without eavesdropping, the frequency of individual aggression approaches 1 when relatedness r is zero and the relative value of the resource $\frac{v}{c}$ approaches 1, since virtually all individuals have the hawk phenotype at these parameter values. Individual aggression drops as relatedness increases and as the relative value of the resource falls (Figures 4.2a and b). Individual aggression is 0 when doves go to fixation, which occurs when $r = 1$ and in the limit as $\frac{v}{c} \to 0$. The frequency of individual aggression is higher with eavesdropping than without, as long as doves are not at fixation, in which case there is no difference (Figure 4.2c).

Escalated aggression. The frequency of escalated aggression is the proportion of encounters in which both players play *hawk*. In our model, a reasonable 'naïve' estimate of the frequency of escalated conflict given the level of individual aggression might be the square of (4.1). This is the level of escalated conflict that would obtain if all players simply chose *hawk* with the probability given by (4.1). The actual frequency of escalated conflict differs from this estimate because the probability that an eavesdropper plays *hawk* is not generally independent of the probability that its opponent also plays *hawk*. The actual expected level of escalated conflict is

$$(x^*)^2 \left(1 - p_E(x^*, y^*)\right)^2 + 2x^*(1 - x^*)y^* \left(1 - p_H(x^*, y^*)\right) + \left((1 - x^*)y^*\right)^2 . \tag{4.2}$$

Similar to individual aggression, the frequency of escalated conflicts approaches 1 when relatedness r is zero and the relative value of the resource $\frac{v}{c}$ approaches 1. Escalated aggression drops as relatedness increases and as the relative value of the resource falls (Figures 4.3a and b). In both cases, escalated conflicts never occur when doves go to fixation in equilibrium. The frequency of escalated conflicts is higher with eavesdropping than without, as long doves are not at fixation in both models, in which case there is no difference (Figure 4.3c).

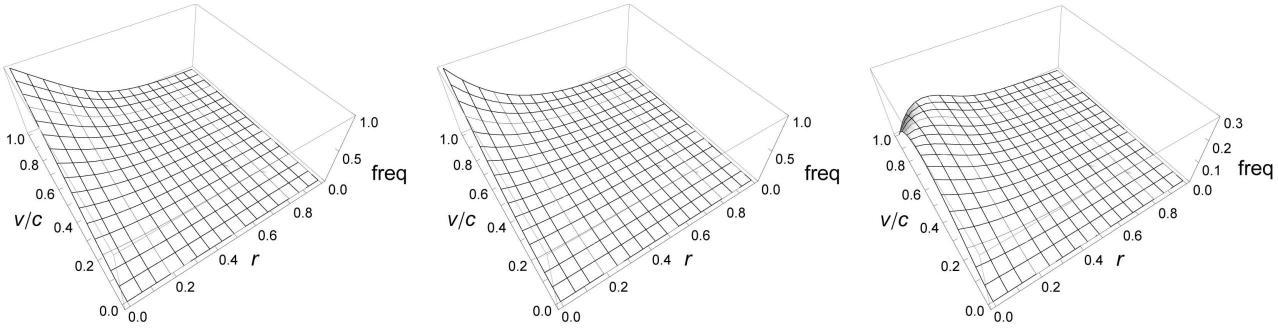

(A) With eavesdropping (B) Without eavesdropping (C) Difference

Figure 4.3. Level of escalated aggression in the continuous strategies hawk-dove game (a) with and (b) without eavesdropping. The frequency of escalated aggression is the expected proportion of *hawk-hawk* encounters in equilibrium. (c) shows the difference in the frequency of escalated aggression between the two models, i.e. with eavesdropping minus without eavesdropping.

The higher frequency of escalated conflict in the eavesdropping model is intuitively consistent with the higher level of individual aggression. This in turn reflects the additional value of winning an encounter, as described by Johnston (2001) – in addition to the payoff from the current encounter, winning also increases the probability of winning against eavesdroppers in future rounds. However, the frequency of escalated conflict given by (4.2) is not generally equal to the square of (4.1) – this is only the case when the population is monomorphic in equilibrium. Johnstone (2001) notes that the frequency of escalated conflict is lower than the square of individual aggression when relatedness is zero. This is also true with positive relatedness in the continuous strategies model, in contrast to the discrete strategies model (Figure 4.4).

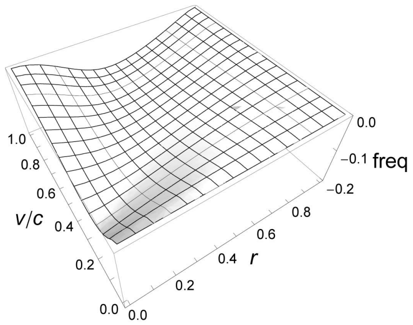

Figure 4.4. Level of escalated aggression in the continuous strategies eavesdropping game less the square of individual aggression. The frequency of escalated aggression is the expected proportion of *hawk*-*hawk* encounters in equilibrium.
